# Surface electromyography: A pilot study in canine spinal muscles

**DOI:** 10.1016/j.mex.2024.103007

**Published:** 2024-10-22

**Authors:** A.M. Ribeiro, D. Pereira, G. Bastos Gaspar, M. Costa dos Santos, H. Plácido da Silva, J.F. Requicha

**Affiliations:** a AniCura Restelo Centro Veterinário, Rehabilitation and Sports Medicine, Rua Gregório Lopes Lote 1524 loja D, 1400-195 Lisbon, Portugal; b Department of Veterinary Sciences, University of Trás-os-Montes e Alto Douro, Quinta de Prados, Apartado 1013, 5001-801 Vila Real, Portugal; c Animal and Veterinary Research Centre (CECAV), Associate Laboratory for Animal and Veterinary Sciences (AL4AnimalS), Quinta de Prados, 5001-801 Vila Real, Portugal; d Instituto Superior Técnico, Department of Bioengineering, Av. Rovisco Pais, 1049-001 Lisbon, Portugal; e Instituto de Telecomunicações, Av. Rovisco Pais, 1049-001 Lisbon, Portugal

**Keywords:** Dog, Intervertebral disc disease, Surface electromyography, Muscle, Motor units, Veterinary rehabilitation, Surface electromyography (sEMG)

## Abstract

In veterinary practice, rehabilitation modalities are often used to help in the recovery of animals affected by InterVertebral Disc Disease (IVDD), a condition frequently observed in chondrodystrophic dog breeds and can lead to Spinal Cord Injury (SCI), resulting in pain, motor impairments and neurological deficits, but there is a lack of objective assessment tools for patient evolution. In this work, an innovative approach using surface ElectroMyoGraphy (sEMG) is proposed to be applied in the field of veterinary medicine rehabilitation. The observed results are thought to be a direct result of nerve compression, leading to unusual patterns of muscle activation; this phenomenon can be attributed to muscle denervation, where the loss of Motor Units (MU) is the primary cause. This is thought to be responsible for the decrease in recorded sEMG amplitude and the increase in frequency observed in the pathological group.

• This study involved rigorous animal preparation and signal acquisition protocols, involving multiple exercises and sub-movements, which were subsequently analysed.
• RMSA is most used metric to analyse amplitude in sEMG signals, as it results in a more representative measurement of the signal variability than the Mean amplitude or the Standard Deviation.

## Graphical abstract

**Figure:**
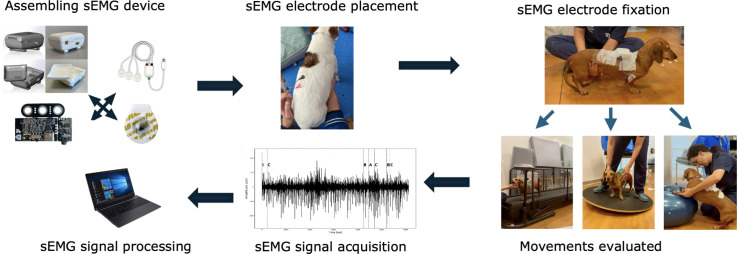

Specifications table

**Table:** 

Subject area:	Veterinary Medicine
More specific subject area:	Neurodiagnostic
Name of your method:	Surface electromyography (sEMG)
Name and reference of original method:	Not applicable
Resource availability:	Not applicable

## Background

InterVertebral Disc Disease (IVDD) is by far the most common cause of Spinal Cord Injury (SCI) in dogs [[1], [2], [3], [4]], usually occurring after extrinsic traumatic forces such as road accidents [[5], [6], [7]]. As in humans, SCI in dogs can have devastating effects on the physical and mental well-being of the individuals affected, but also for their family [[1], [8], [9], [10]]. Chondrodystrophic breeds such as the Dachshund, Beagle, French Bulldog and Pekingese are much more likely to develop disc herniation than non-chondrodystrophic breeds [[1], [3], [4], [9], [10]], initial degenerative changes are completed as early as one year of age [[1], [8], [11], [12]]. Approximately 75 % of intervertebral disc herniations in these breeds are found at the level of the thoracic vertebra 12 (T12) to lombar vertebra 2 (L2) [13,14,7].

To the best of our knowledge, there are few objective measurement tools available for use in Veterinary Rehabilitation Medicine to assess patient improvement during treatments, especially in the neurological patient. Valuable tools for assessing neuromuscular disorders have been emerging, particularly surface ElectroMyoGraphy (EMG) [[5], [15], [16]] which measures and analyse the electrical activity occurring in muscle fibres [5,6,17,18]. This technique allows the evaluation of important information such as shape, duration, phases, and amplitude of electric impulses, which helps to identify pathological conditions [5,17], including chronic denervation or fasciculation, even in muscles that appear normal during a clinical examination.

While sEMG parameters have been standardized for human use [[15], [19]], the recent in-depth studies in animals are inconclusive about their validity in veterinary practice [20,21]. The lack of standard procedures and references that are necessary for the validity, reproducibility, and quality of the results, represents a limitation that extends to every variable of sEMG, such as the electrode's materials, dimensions and placement, the Inter-Electrode Distance (IED), skin preparation procedures, and the chosen exercises to be measured [21]. Most of the previous literature on animal sEMG primarily focuses on locomotion muscles in horses and dogs, specifically limb muscles, due to their active role, well-defined insertions, and minimal skin displacement, which enhances signal quality [[21], [22], [23]].

With this work, the authors aimed to explore the following research questions:

– RQ1: Is it possible to devise a device suitable for sEMG data acquisition from chondrodystrophic breeds while performing static and dynamic activities?
– RQ2: What experimental setup and protocol can be used to evaluate neuromuscular function?
– RQ3: Can sEMG be an added value tool to assess neuromuscular function in Dachshund dog's thoracolumbar spinal region, considering that this is a highly susceptible area to intervertebral disc disease?

## Method details

### Pre-testing

For this study, four miniature Dachshunds aged between 3 and 7 years old attended at AniCura Restelo Veterinary Rehabilitation Centre were included (Table 1), upon an informed consent by the owners. A comprehensive orthopaedic and neurologic examinations allowed to confirm that two animals (number 1 and 2) had no clinical signs of neurologic or orthopaedic diseases, and two (number 3 and 4) had IntraVertebral Disc Disease (IVDD) confirmed by CT scan. The later individuals were then subjected to surgery to decompress spinal cord (hemilaminectomy) followed rehabilitation sessions.

**Table 1:** Information about the four studied dogs.

Animal	Breed	Age (years)	Sex	Diagnosis	Outcome
1	Mini bristle-haired Teckel	5	Female	No sign of IVDD	Not applicable
2	Mini long-haired Teckel	3	Female	No sign of IVDD	Not applicable
3	Short-haired Teckel	7	Female	IVDD in T11-T12-T13	Recovery of about 3 months of physiotherapy – independent ambulation
4	Short-haired Teckel	7	Male	IVDD in T11-T12 -T13	No deep pain sensation, nonambulatory

### Assembly of the surface ElectroMyoGraphy (sEMG) Device

**Fig. 1:**
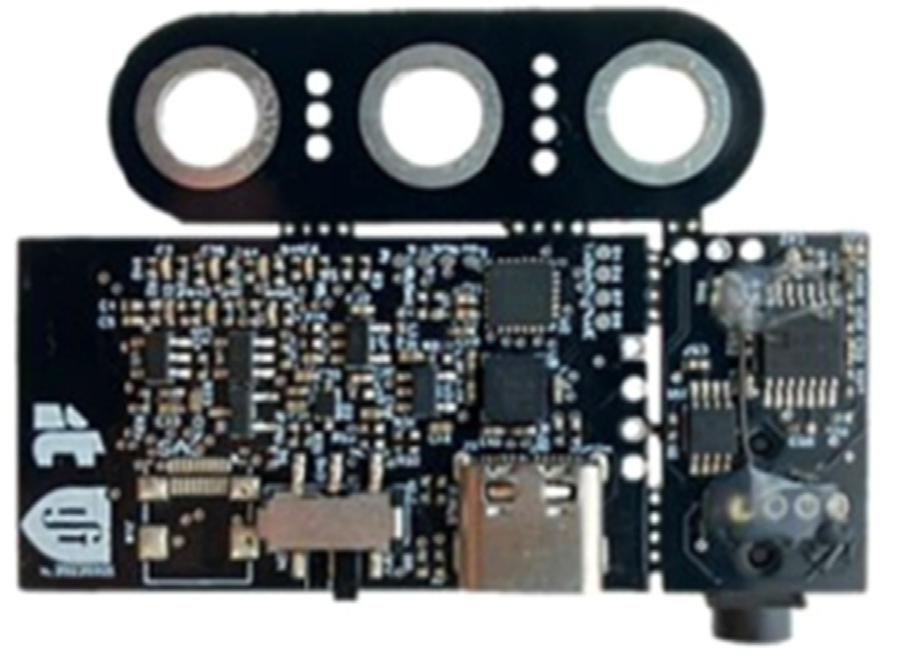
ScientISST device.

**Fig. 2:**
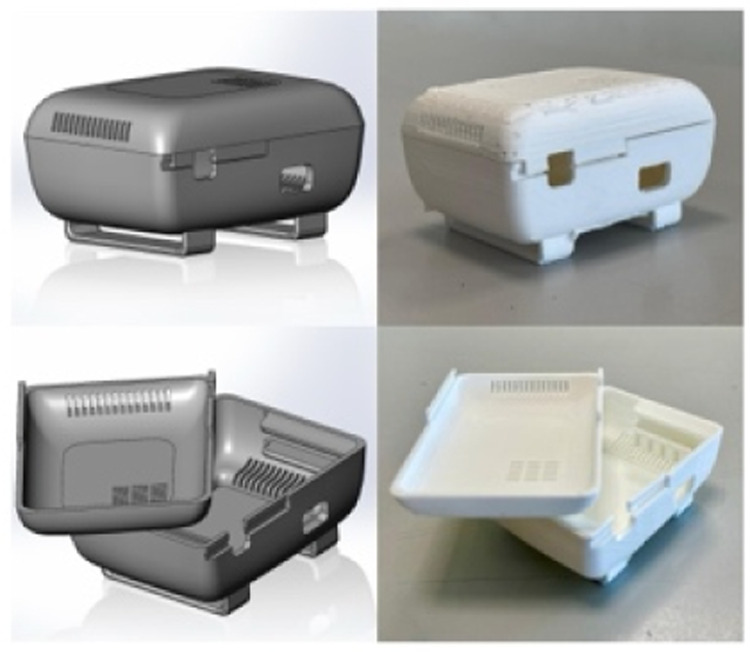
3D model (left) and printed case (right).

In this work, a ScientISST^Ⓡ^ device was modified so that the onboard sensor uses a passing band of 10 to 400 Hz (Fig. 1) and has a gain of 1000. It included three electrodes, two differential leads (positive and negative) and a reference lead, as well as a 3-axis accelerometer for motion sensing. Both these sensors were sampled at a rate of 1000 Hz. The Analog-to-Digital Converter (ADC) has a 12-bit resolution and works at 3.3 V, making it possible to for the sEMG sensor to measure voltages between 0 and 3.3 mV, with a 0.81 µV amplitude resolution. For the sEMG sensor to be more easily held and protected, a small enclosure was designed and 3D-printed (Fig. 2). The overall result addresses our RQ1.

### Preparation of the skin, electrode placing and sEMG device mounting

**Fig. 3:**
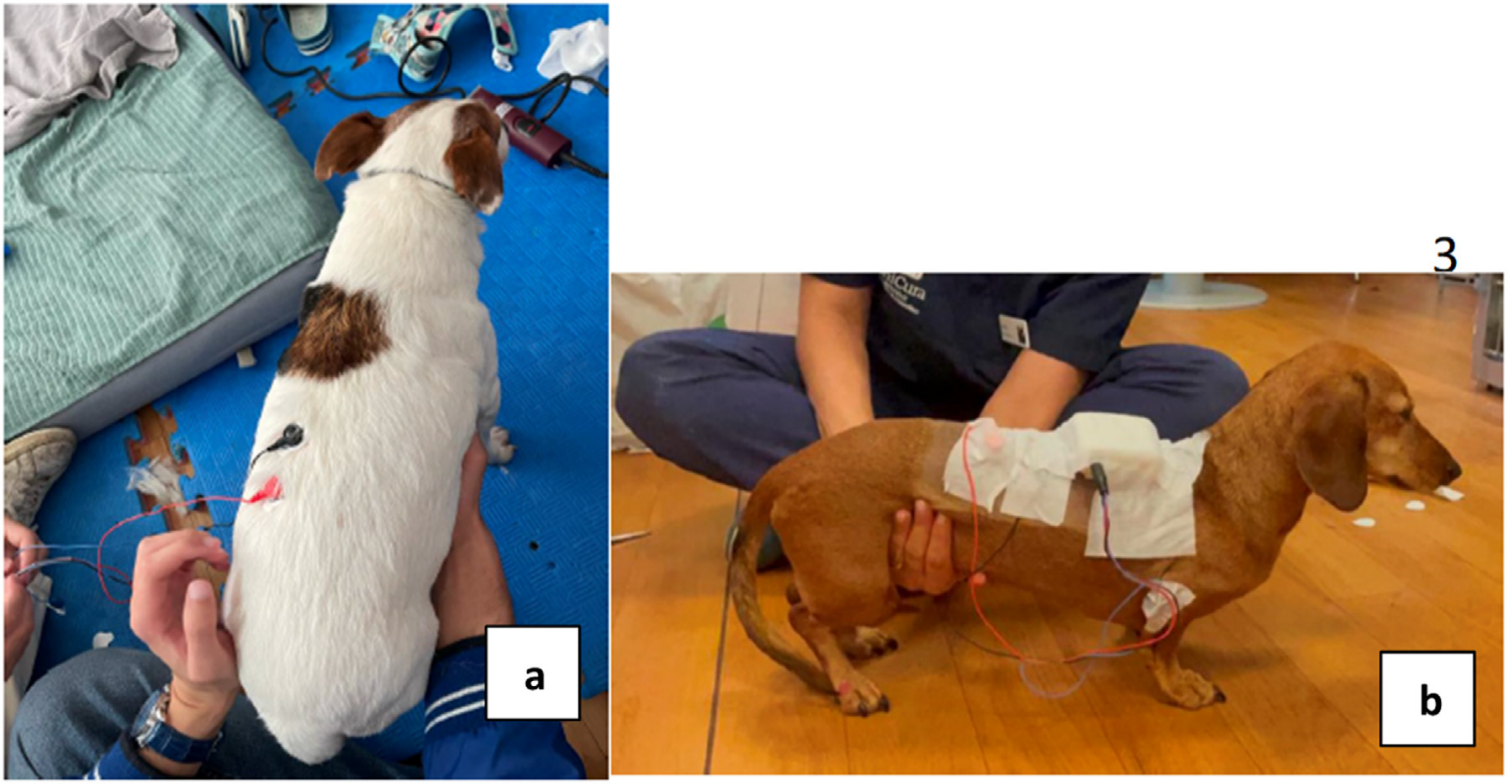
Animal preparation depicting (a) Electrodes placement, and (b) Cohesive bandage fixation.

Before the electrode's application, dogs’ hair was clipped in three small 1.5 × 1.5 cm areas, square shape, two in the paraspinal region between thoracic T12 (active lead - positive) and lumbar L2 (ground lead - negative) vertebrae (Fig. 3a), 1 cm apart, and one in the medial aspect of the elbow (reference lead). These areas were then cleaned with alcohol to remove grease and to diminish the impedance, which could affect the muscle activity recorded by the electrodes. The sensor was then fixed on to the animal's torso, with an adessive retention bandage (10 cm ×10 m Omnifix E, Hartmann, South Africa), allowing the three leads to be connected to the anatomical sites of interest (Fig. 3b). Upon mounting, the dogs were allowed to get used to the new object before the signal acquisition began.

### Movements protocol

**Fig. 4:**
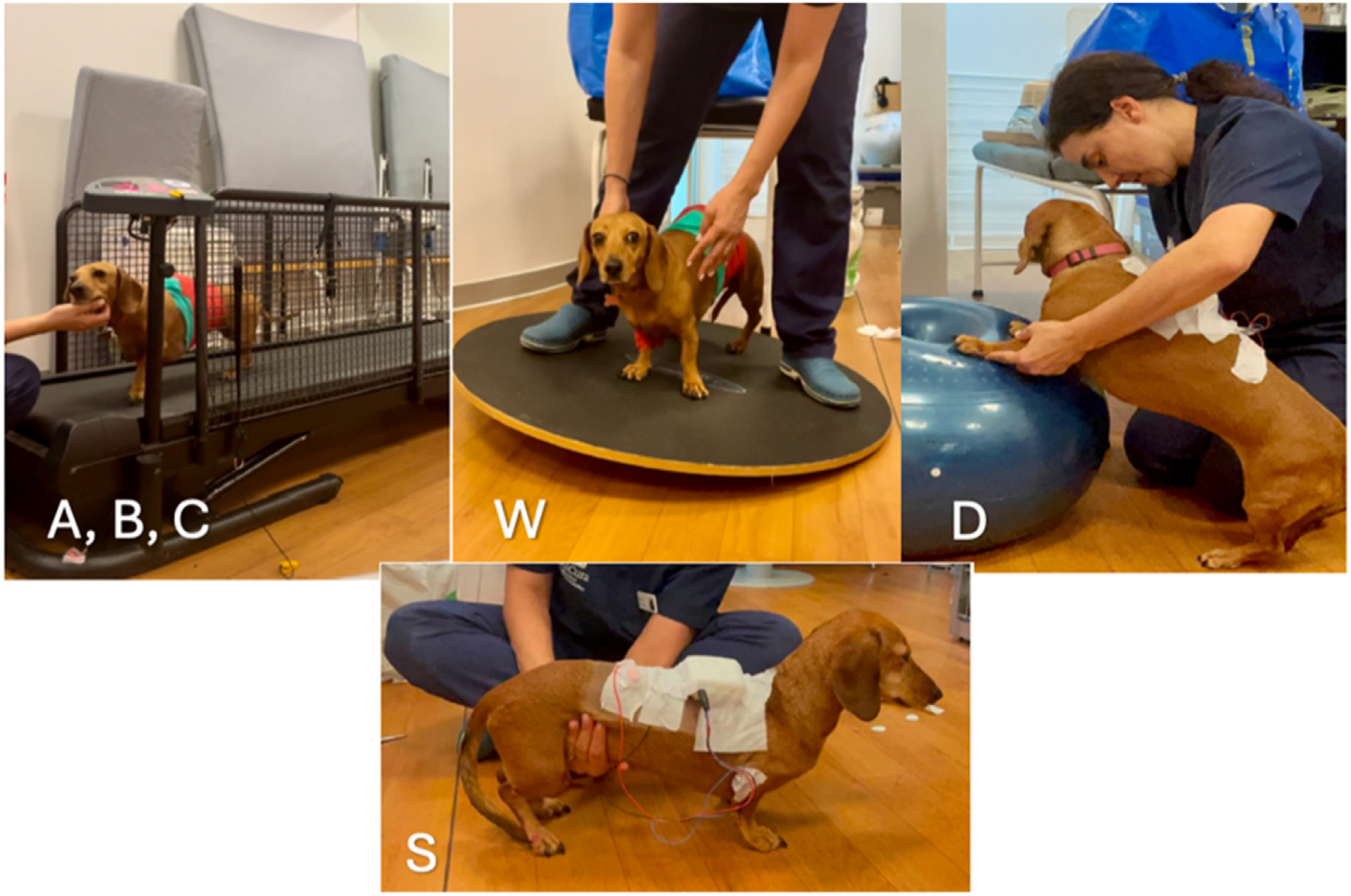
Movements evaluated with sEMG: walking on a treadmill - accelerating (A), decelerating (B) and stable walking (C); balancing on a Wobble board (W); and isometric position on a donut ball (D) and stationary standing up (S).

In response to our RQ2, the protocol devised to evaluate muscle activity consisted of the following activities: (A,B,C) walking on a treadmill at 0.7 miles per hour, this task was divided in tree sub-movements - accelerating, decelerating and stable walking); (W), balancing on an oscillating Wobble board; and (D) isometric position with front paws on a donut ball and (S) stationary standing). The duration of each exercise varied slightly but was ensured to surpass the 1-minute threshold (Fig. 4).

### Signal acquisition

The signal acquisition was done over Bluetooth, with the data being transmitted in real time data between the device and a laptop computer. The exercises performed were accompanied with video recording, for later characterization of the signal based on the dog's behaviour. For this, a camera mounted on a tripod was placed in front of the dog, while the device was simultaneously streaming data to the laptop computer. To facilitate the synchronization between the video and the signal post-processed, an audio cue was made by the computer to indicate the acquisition had begun. This sound was audible in the video, marking the moment from which the video should be analysed.

### Signal processing

Each of the sEMG signals was filtered using a 6th order band-pass digital filter with a band of [24, 500] Hz, to narrow the spectrum of the signal to the frequencies of interest for an EMG [25], while also complying with the Nyquist–Shannon Sampling Theorem [26]. In addition, a notch filter with cut-off frequency of 50 Hz was also employed. This filter is like a band-stop filter with a very narrow band, allowing for the partial removal of powerline noise from ECG signal crosstalk, which was observed in the raw EMG signal, since the canine ECG encompasses frequencies around 50 Hz as indicated by Xu et al. [27]. The Mean and Root Mean Square Amplitude (RMSA) were extracted from the absolute sEMG values, while the Standard Deviation (σ) was calculated using the signal as-is. The standard deviation is calculated using equation:

(1) $$\begin{array}{c} Mean\;Amplitude=\frac{1}{N}\sum_{i=1}^{N}x_{i}\\ RMSA=\sqrt{\frac{1}{N}\sum_{i=1}^{N}x_{i}^{2}} \end{array}$$

(2) $$\sigma =\sqrt{\frac{1}{N}\sum_{i=1}^{N}{\left(x_{i}-\mu \right)}^{2}}$$

A spectral analysis was also done, by means of a Fourier Transform. Considering only the relative power of each frequency ranging from 0 Hz to 500 Hz, calculated through the absolute values of the square of the Fourier transform. From there, the area under the curve was calculated (Power Integral). A normalized power spectrum was then derived (re-scaling the values so the area under the curve is unitary), which in turn allowed the comparison between different sub-movements, and the extraction of the maximum, mean and median frequencies (F_max_, F_mean_, and F_median_, respectively). All the signal-processing steps within the subsection were performed using a custom Python code base.

### Method validation

**Fig. 5:**
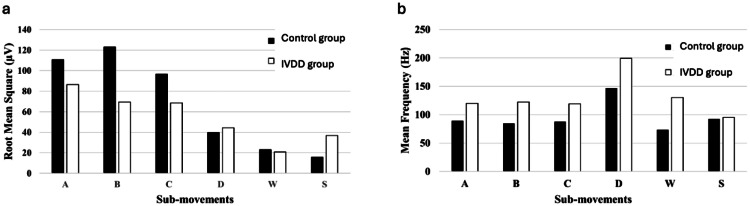
Root Mean Square (a) and Median Frequency (b) value comparison between Control and IVDD Groups.

To conduct a comprehensive quantitative assessment, the procedures outlined in the methods section were executed, and the resulting information for each tested canine, categorized into the respective sub-movement activities, is presented in the following table (Table 2). Additionally, to ensure a more precise and easily interpretable analysis of the data, the information provided in the Table 2 is visually represented in the form of a column graph (Fig. 5).

**Table 2:** Features regarding sEMG measurements on the animals from two experimental groups. Legend: A – Accelerating, treadmill; B – Decelerating, treadmill; C – Stable walking, treadmill; D – Donut ball (isostatic); W – Wobbler board; S – Standing still (isostatic).

Movement	Mean (µV)	Standard deviation (µV)	rms (µV)	integralP	Fmax	Fmean	Fmedian
**Control Group**							
**A**	94.50	111.67	110.68	11,078,822.94	25.07	89.65	69.80
**B**	16.49	24.34	22.97	298,982.79	11.72	73.40	19.89
**C**	103.31	124.33	123.10	12,476,849.53	18.20	84.86	59.78
**D**	38.30	42.11	39.77	985,165.67	35.68	146.85	126.41
**W**	80.04	97.39	96.47	9,087,433.78	15.27	88.07	63.68
**S**	14.86	16.05	15.68	148,987.18	20.15	92.88	68.06
**Intravertebral disc disease Group**							
**A**	77.85	85.92	86.38	3,696,735.28	15.44	119.95	79.33
**B**	18.75	20.74	20.75	216,391.90	12.57	130.37	57.58
**C**	59.61	69.90	69.55	2,540,061.67	15.12	122.39	83.59
**D**	44.16	44.35	44.41	985,880.35	15.27	199.13	185.61
**W**	59.39	68.67	68.61	2,363,519.46	14.80	119.00	84.00
**S**	35.24	38.03	36.76	845,546.06	17.88	95.25	23.43

The charts in Fig. 5 represent the comparison between the two subject groups regarding RMSA (Fig. 5a) and F_mean_ (Fig. 5b) values for each of the different sub-movements.

Regarding the RMSA parameter, the bar chart (Fig. 5a) the control group revealed substantially higher values in the treadmill exercise (sub-movements A, B and C). There was also a significant divergence between control and IVDD subjects, in which the control subjects reported higher values. In contrast, the isostatic tasks suggest the opposite trend, although with a less pronounced difference between the two groups. This effect is especially prominent in the Wobbler board exercise (W), in which no significant discrepancy was found. When examining the mean frequency feature, a more consistent pattern emerged during the analysis. Across all the sub-movements examined, the IVDD group consistently excelled over the control group. The three sub-movements related to the treadmill exercise revealed nearly constant values within each group, resulting in a nearly constant difference as well. Furthermore, the sub-movement D registered the highest values for either one of the groups. Focusing on the disparity between the control and IVDD subjects, the larger gap was exhibited in the Wobbler movement, whereas the slimmest was in the standing still segments.

When examining the mean frequency feature (Fig. 5b), a more consistent pattern emerged during the analysis. Across all the sub-movements examined, the pathological group consistently excelled over the healthier group. The three sub-movements related to the treadmill exercise revealed nearly constant values within each group, resulting in a nearly constant difference as well. Furthermore, the sub-movement D registered the highest values for either one of the groups. Focusing on the disparity between the healthy and pathological subjects, the larger gap was exhibited in the Wobbler movement, whereas the slimmest was in the standing still segments.

Based on Lowery and O'Malley [28] and Petrofsky [29], RMSA is most used metric to analyse amplitude in sEMG signals, as it results in a more representative measurement of the signal variability than the Mean amplitude or the Standard Deviation, for instance. Regarding the frequency domain, the Mean Frequency (F_mean_) varies between each group (“Control” and “IVDD”) in the same way the Median Frequency (F_median_) does and is less susceptible to noise than the Maximum Frequency (F_max_). Therefore, RMSA and F_mean_ were chosen to be further evaluated and compared. As far as the Power Integral is concerned, the presented feature exhibited results that lacked relevance for the proposed work.

Neuromuscular disorders, such as nerve compression due to IVDD can lead to variations in muscle function and lower sEMG amplitudes, due to abnormal muscle activation patterns leading to denervation [[23], [30], [31], [32]], the affected muscles don't receive the necessary neural stimulation for normal contraction and neurogenic atrophy occurs. A sustained denervation in the muscle causes the loss of Motor Units (MU), which are the muscular areas enervated by a single motor neuron. This loss is thought to result in a reduction of the recorded sEMG amplitude, as fewer MU are being stimulated, which is associated with a neuropathologic condition [[30], [31], [32]]. Like the variations shown in the amplitude, other discrepancies, such as in frequency, can be explained by nerve damage, neuropathies, which may be caused by nerve compression, can accelerate muscle fatigue, which will affect the sEMG signal characteristics [33]. Initially, as a muscle fatigues, there might be an increase in sEMG frequency; contrarily, as fatigue progresses, the sEMG amplitude tends to increase while the frequency may decrease [33]. However, the exercises approached in this experiment were short in duration and not physically demanding, preventing late-stage fatigue. Another consequence of denervation is often muscle fibrillation, resulting in very small and erratic electrical impulses in individual muscle fibbers. These abnormal firing patterns of motor units can lead to higher sEMG frequencies [[32], [34]]. These effects support the data extracted from this pilot study (Table 2 and Fig. 5), which suggest a decrease in sEMG amplitude and an increase in sEMG frequency in exercises featuring more dynamic muscle activation (i.e. A, B, C and W). Overall, results answer our RQ3.

Future steps could be taken into consideration regarding the simultaneous motion data analysis. In addition to acquiring the sEMG signal, 3-axis accelerometer data is also recorded; in the future, this could be used to perform a biomechanical assessment of body parts of interest, providing further detail into the effects of IVDD in mobility and muscle activity. These future improvements and features, paired with the testing techniques and protocols devised in this study, would culminate in the creation of an improved device. Such a device would be better fit for clinical use, facilitating the non-intrusive use of sEMG-based methods for patient monitoring in veterinary rehabilitation medicine.

## Limitations

One of the difficulties we encountered during this study was preparing the skin and keeping the electrodes in the correct place, as well as the reluctance of owners to cut their animals' hair. For this reason, the authors believe that exploring dry electrodes adapted to the individualities of veterinary medicine would be fundamental to capture the signal more easily and without implying aesthetic changes to the animals' hair, which is one of our objectives in future studies.

As aforementioned the obtained findings were derived exclusively from a sample which consisted of two animals for each experimental group. Consequently, it is imperative that subsequent investigation be undertaken on a more extensive scale to discover statistically significant data relating to Dachshunds with both healthy controls and with IVDE. While the number of subjects in this experiment was not sufficient to provide a statistically valid and generalized assertion regarding the effects of IVDH on the sEMG signal, the results suggest a potential trend worth further investigating. The authors would also like to point out, that a new electrode design, one that wouldn't need clipping of the subject fur would be beneficial, as acceptance by animal owners to enter any research program would be greater.

### Ethics statements

This pilot study was conducted with four dachshund dogs, from AniCura Restelo Veterinary Center and AniCura Restelo Veterinary Hospita, who were already visiting for routine consults and rehabilitation treatments. No invasive procedures were involved. The study took place within a clinical context, where procedure had a clear clinical indication. Owners provided written consent form, having been fully briefed on each step of the process, and were present during signal acquisition. Consequently, formal ethical approval was not deemed necessary.

## CRediT author statement

A.M.R designed the protocol, prepared the animals before electrode placement, placed electrodes and sensor, conducted the exercises, and prepared the manuscript. D.R., G.B.G. and M.C.S. designed the sEMG sensor and protective case, were responsible for the signal acquisition, segmentation and processing and prepared the manuscript. H.P.S. and J.F.R. supervised the work, revised the protocol, and prepared the manuscript. All authors have read and agreed to the published version of the manuscript.

## Declaration of competing interest

The authors declare that they have no known competing financial interests or personal relationships that could have appeared to influence the work reported in this paper.

## Data Availability

Data will be made available on request.
